# Continuing education for the chiropractic profession: a cross-sectional study analyzing potential barriers to future chiropractic academic and research development

**DOI:** 10.1186/s12998-025-00596-x

**Published:** 2025-08-21

**Authors:** Shannon Schueren, Dean L. Smith, Christopher A. Malaya, Jeffrey A. King, Nathan D. Schilaty

**Affiliations:** 1 Department of Kinesiology, University of Georgia, Athens, GA USA; 2 Department of Kinesiology, Nutrition and Health, Miami University, Oxford, OH USA; 3 Essence of Wellness Chiropractic Center, Eaton, OH USA; 4 Teaching, Learning, and Information Resources, Parker University, Dallas, TX USA; 5 Department of Neurosurgery, Medical College of Wisconsin, Milwaukee, WI USA; 6 Department of Neurosurgery, Brain, & Spine, University of South Florida, Tampa, FL USA; 7 Center for Neuromusculoskeletal Research, University of South Florida, Tampa, FL USA

**Keywords:** Continuing education, Chiropractic, Licensure, Research, Teaching, Post-graduate

## Abstract

**Background:**

Continuing education (CE) for chiropractors is mandated by state licensing boards to ensure ongoing learning and to maintain professional excellence. While incorporating research into CE programs is crucial for practitioners to remain dynamic and evidence-based, conducting research and academic pursuits is necessary for further development of the profession. We hypothesized that fewer U.S. states would provide CE credit for the completion of research or higher-education teaching activities within the chiropractic profession compared to other health professions.

**Methods:**

Internet searches of publicly available state board websites for each profession was undertaken with a cross-sectional study design between 12/19/2024 and 03/01/2025. Data extraction focused on whether CE was granted for research (publication and/or peer review) as well as academic pursuits (higher education and/or CE instruction). Descriptive statistics determined the frequency of states allowing CE while Fisher’s Exact test and one-way ANOVA was performed to compare states granting CE credits for research and teaching as well as comparisons among the professions.

**Results:**

Only 16 US states allow DCs to claim research activities for CE credit while 50% allow teaching for CE credit. This is significantly lower (*p* < 0.001) than ATs, DOs, and MDs where teaching and research activities are accepted for CE credit in all states.

**Conclusions:**

Precluding research and higher-education teaching opportunities for CE presents a potential barrier to chiropractic academic and research development at present and in the future.

**Supplementary Information:**

The online version contains supplementary material available at 10.1186/s12998-025-00596-x.

## Introduction

In the United States (US), chiropractic licensure is overseen at the state level with 52 unique and individual sets of statutes and administrative rules (i.e., 50 states, District of Columbia, and territory of Puerto Rico; hereby all referred to as ‘state(s)’) [1]. Maintenance of licensure is dependent on licensed chiropractors (DCs) taking approved continuing education (CE) courses and/or completing approved activities to obtain the required CE hours or credits within the renewal period, typically over a 1–2 year period, depending on the state [1]. If the CE requirements are not successfully achieved within the allotted time frame, licensees may face suspension, revocation, penalties, or fines [1].

Approval of chiropractic CE hours or credits is dependent on a range of requirements that vary by state. Typically, these requirements emphasize the host of the course be on a list of approved providers, the speaker or instructor should have the appropriate experience, credentials, and subject matter expertise to teach the course, the content must be relevant to the practice of chiropractic, and the course should include clearly defined learning objectives for attendees [2]. Depending on state rules and laws, CE hours or credits can be obtained, in person, with distance learning, or through teaching CE courses [3]. However, many states require chiropractic CE credits to be approved by the Council of Chiropractic Education (CCE) or Providers of Approved Chiropractic Education (PACE).

Medical doctors (MDs) and osteopathic doctors (DOs) undergo rigorous residency training upon the completion of their degree programs wherein they are able to accumulate CE credit for all or part of their training hours [4, 5]. Conversely, chiropractors have access to optional residency programs, but currently only four states accept residencies or fellowships for CE credit [6], a demonstration of how the chiropractic profession is trailing behind other medical professions. Stuber et al. [3] found that the majority of chiropractors have interest in completing further training, such as a Master’s degree, particularly if the academic program would count towards continuing education credits. This implies that chiropractors may perceive a barrier to further academic pursuits, including research, due in part to the lack of understanding and acceptance by their state licensing board for such professional development. While there has been a substantial growth in the number and quality of chiropractic research publications over the past 50 years [7], there is a shortage of chiropractic clinicians who have the experience and training to conduct clinical research [8]. Thus, with the current barriers to CE regarding academic and research pursuits, the profession’s growth capacity may be further impeded if an academic/research CE barrier is verified.

It is unclear how CE credits for research and higher-education teaching activities in the chiropractic profession compare to other similar professions, including MDs, DOs, physical therapists (PTs), and athletic trainers (ATs). These professions, other than PTs, have national standards for CE credits which include recognizing research and academic teaching as qualified CE [4, 5, 9]. This appears to be in line with the National Academy of Medicine’s purpose of continuing medical education, which is to both reinforce current practice as well as translate new knowledge into practice [10]. However, a lack of similar alignment of the chiropractic profession could significantly impede knowledge development and adoption of evidence-based guidelines. Therefore, the aim of this study was to describe which states allow chiropractors to claim CE credits/hours through higher education teaching, publication, or participation in the peer review process. We hypothesized that fewer U.S. states would recognize research or higher education teaching activities as eligible for CE credit within the chiropractic profession compared to other health professions.

## Methods

This study followed the *Strengthening the Reporting of Observational Studies in Epidemiology* (STROBE) guidelines [11]. This study did not qualify as human subject research and, therefore, did not require Institutional Review Board (IRB) approval [12]. The cross-sectional design of this study was based on and modeled from prior research that examined chiropractic state board websites for similar information [6, 13–15].

### Data recording, organization, and validation

Data were recorded in an Google spreadsheet (Google LLC, Mountain View, CA). Between December 19th, 2024 and March 1st, 2025, five investigators (SS, DLS, CAM, JAK, NDS) recorded CE and licensure-related data from chiropractic board and licensure websites for all 50 states, the District of Columbia, and the US territory of Puerto Rico. The official website for each chiropractic board and/or state legislative website was accessed and reviewed. From the homepage, links to CE, license renewal, and frequently asked question (FAQ) sections (if available) were searched manually for content related to research and academic credit in addition to other variables (see additional files). Administrative code, where available, was also queried for descriptions related to CE credit offered for research and academic hours. Variables entered into the spreadsheet included: official name of the Board; Board statute oversight; process for rule change; CE hours required per cycle; renewal term in years; per year equivalent number of CE hours; whether CE must be board approved or CCE/PACE approved; whether CE courses were chiropractic specific or only to be taught by chiropractors; PACE allowed; number of credits allowed for online CE; percentage of CE credits allowed to be taken online; whether research related activities (manuscript publication and/or scientific peer-review) count toward CE; whether academic related activities (university level teaching or CE instruction) count toward CE; allowed CE topics; whether academic or research related CE credit information was found in FAQ sections. For the purpose of this paper, university level teaching is defined as an accredited higher education institution.

For the comparator professions (MDs, DOs, ATs, and PTs) state board required credit hours were obtained from each state’s legislative website for each profession and documented within a spreadsheet. Accepted CE credit types are nationally standardized for MDs, DOs, and ATs through the Accreditation Council for Continuing Medical Education and American Medical Association (ACCME and AMA; accme.org, ama.org), American Osteopathic Association (AOA; osteopathic.org) and Board of Certification for Athletic Trainers (BOC-ATC; BOCATC.org) respectively. These national standardized CE requirements include varying categories of CE such as Physician’s recognition awards (PRA) Category I/II (MDs), Category Ia/Ib/IIa/IIb (DOs), and Category A-D (ATs). For PTs, accepted CE credit types differ at the state level, similar to chiropractic. The official website for each physical therapy board and/or state legislative website was accessed and reviewed for allowance of research activities or teaching as accepted forms of CE credit was documented.

The 5 investigators served as the initial data extractor for approximately 10 states each. Following initial data extraction, each investigator verified the data extraction of a fellow investigator. Any ambiguities or discrepancies in data extraction that could not be resolved between two investigators were adjudicated by a 3rd investigator. These ambiguities were uncommon and usually involved information that was not provided in the CE portion of the state chiropractic board’s website. For pragmatic reasons, when no explicit information regarding academic or research related CE could be found on the board’s website or through administrative code, that state was classified as offering no CE for those activities. During data extraction, four categories were created to classify whether research-related activities (e.g., publishing in an indexed, peer reviewed journal or performing peer review for an indexed peer reviewed journal) and/or academic related activities (e.g., higher-education instructor/professor, verified participation as an instructor of CE) counted toward CE credit requirements. These categories were chosen as they relate to the specific interest of this manuscript.

### Data analysis

Data was exported into *JMP 18 Pro* for statistical analysis. Descriptive statistics were determined. Fisher’s Exact test was performed to compare the number of states granting CE credits for research and teaching and One-way ANOVA was performed to compare among professions with Tukey’s HSD post hoc comparison performed for each pair when appropriate. An a priori value of ɑ < 0.05 was set for significance.

## Results

**Fig. 1:**
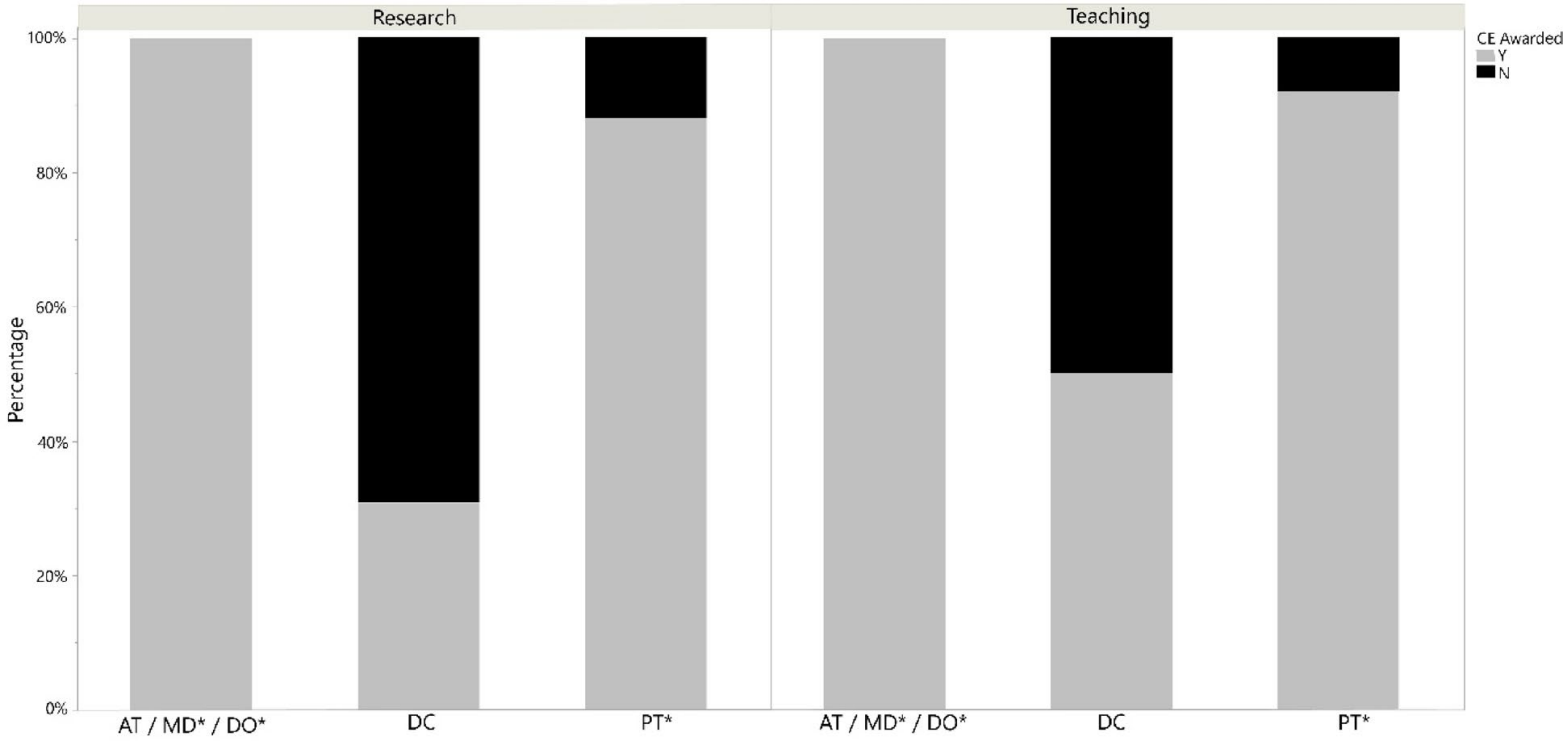
Percentage of states awarding CE credits for teaching and research by profession*.* *excluded states that have no CE requirements (MD/DO: CO, IN, MT, SD, NY; PT: ME) or laws could not be found (PT: PR)

Fisher’s Exact test by profession demonstrated that the number of states allowing CE credit for performance of research were different for DCs compared to ATs, DOs, MDs, and PTs with 69.2% not allowing research CE (*p* < 0.001; Fig. 1). PTs also had lower acceptance of research than ATs, DOs, and MDs with 12% of states not allowing research CE (*p* = 0.012). Requirements for obtaining CE credit for peer-review publication differed by professional body. For MDs, CE credit is only awarded to first authors, while ATs can obtain credit for being first or second author [4, 9]. The AOA does not explicitly state which authors can obtain credit for the DO profession [5]. Regulations varied by state for the DC profession. States that allow for CE credit for performance of university level teaching or instruction of CE courses showed the DC profession was lower from all of the other professions (ATs, DOs, MDs, and PTs) with only 50% allowing for CE for teaching (*p* < 0.001; Fig. 1).

**Fig. 2:**
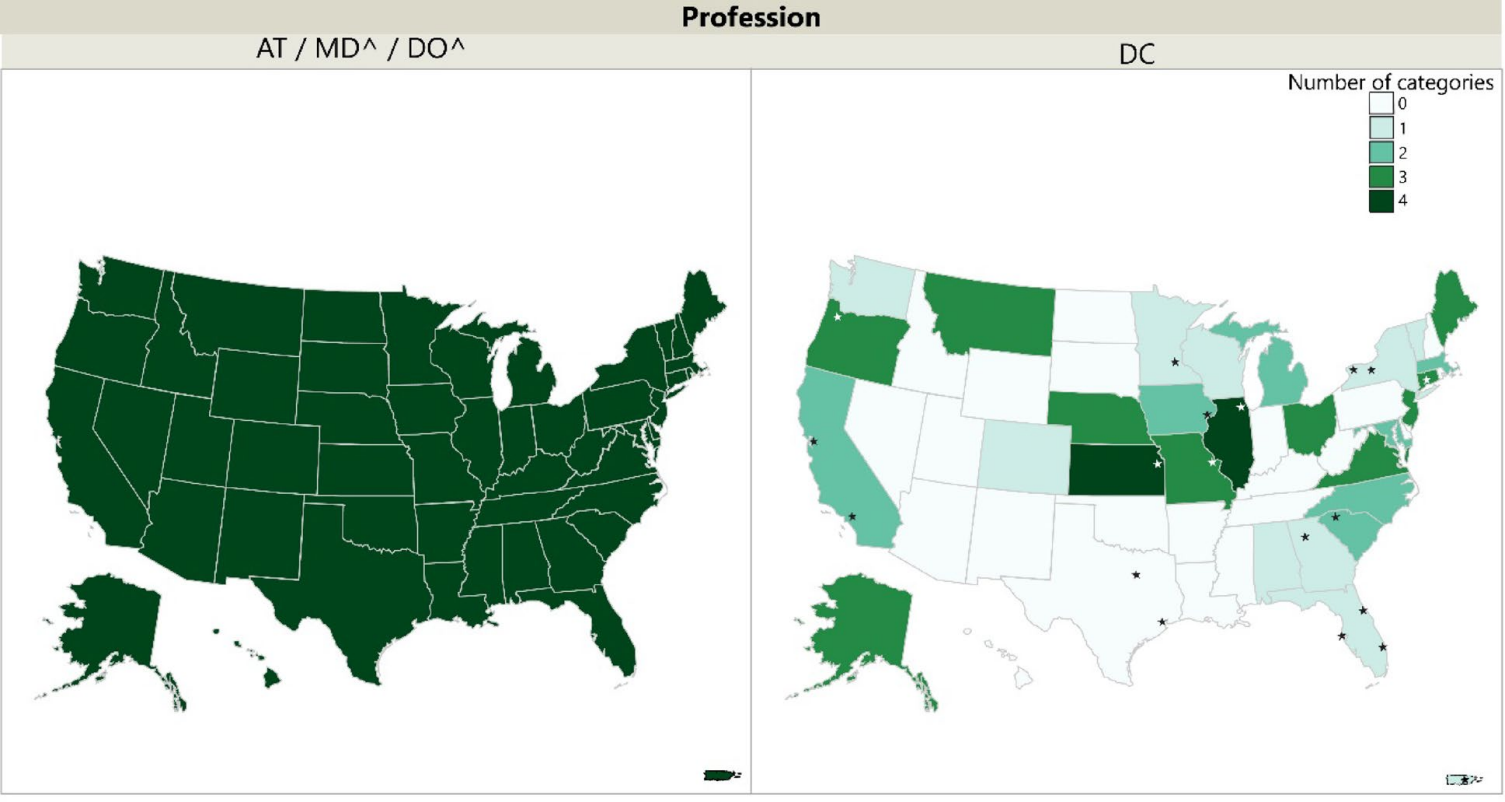
Number of categories in research and teaching awarded CE by state and profession. **^**excluded states that have no CE requirements (MD/DO: CO, IN, MT, SD, NY; PT: ME) NOTE: Stars within the DC map denote chiropractic colleges/universities within the United States and Puerto Rico

In the four categories analyzed, CE credit could be obtained via scientific publication, scientific peer review, university level teaching, and seminar instruction for ATs, MDs, and DOs [4, 5, 9]. For DCs, only 2 states, Illinois and Kansas, awarded CE credit for all four of these categories, while 19 states did not award CE credits for any of these categories (Fig. 2). The PT profession was not separated into specific research or academic categories, and is therefore not included within the below figure.

One-way ANOVA demonstrated significant differences in annual CE hours required by each profession within the five professions observed (*p* < 0.001). Specifically, the PT profession had the lowest annual CE requirement with an average of 14.5 CE hours. PTs were lower than DCs (*p* = 0.010) and the other 3 professions (AT, DO, MD; *p* < 0.001). DCs were similarly lower than these three professions (AT, DO, MD; *p* < 0.001) with an average of 18.5 CE hours required annually. There were no other differences of annual CE hours required by the remaining professions, with ATs, MDs, and DOs requiring an average of 25, 26.7, and 27.7 CE hours, respectively (Table 1).

**Table 1:** Continuing education credits by profession

Profession	Mean CE Hours (95% CI)	% States allow research CE	% States allow teaching CE
AT	25 (22.9, 27.1)	100	100
DC	18.5 (16.3, 20.6)	30.8	50.0
PT*	14.5 (12.3, 16.6)	88.0	92.0
DO*	27.7 (25.4, 29.9)	100	100
MD*	26.7 (24.4, 28.9)	100	100

^*^Excluded states that have no CE requirements MD/DO: CO, IN, MT, SD, NY; PT: ME, PR (specified laws could not be found). AT = athletic training; DC = chiropractic; PT = physical therapy; DO = osteopathic; MD = medicine

Information regarding the specific professions’ acceptance of research and academic work for CE credit and state required CE hours are presented in Additional Files 1–3. A full representation of additional data obtained for DCs (e.g., board rules, regulations, specific state legislative requirements) is presented in Additional File 4.

## Discussion

The chiropractic profession was found to have the least incentives to engage in research and higher-education teaching activities for CE credit relative to the other health professions. All states allow ATs, DOs, and MDs to use research and higher-education teaching towards their customary CE requirements. PTs were found to require the least CE hours for re-licensure, followed by chiropractors, but 92% of states allow PTs to perform research or academic-related CE hours. Illinois and Kansas were the only two states that allow CE credit for all four categories queried: research publication, peer-review, and university level/CE instruction. As such, we think it important to highlight their respective state legislations. Both states have administrative codes that require DCs to adhere to the same administrative code as MDs and DOs [16, 17].

Previous survey research [3] found that DCs have interest in pursuing further academic and research related interests, particularly if that higher education program would count towards CE. We have verified that an academic/research CE barrier does exist for DCs, but not for the other professions. We infer that this barrier is likely hindering the research capacity of the profession which is further constrained by the existing shortage of DCs who have the requisite experience and training to conduct clinical research [8].

While there has been substantial growth in the number and quality of chiropractic research publications over time [7], the fact that the majority of states do not allow research activities to count toward CE impedes the advancement of future research. With the rapid proliferation of chiropractic and health literature, it is more important than ever for chiropractors to stay up-to-date with the latest research. With roughly 82% of chiropractors working within a chiropractic office [18], offering CE credit for the conduct of and participation in research studies would provide a facile means towards additional, clinician-focused projects, as well as further practice-based research and training opportunities.

CE that is grounded in evidence-based data is a means to provide skill and knowledge promotion among practicing clinicians [19]. However, Lyu and Li [19] also suggested that the large, increasing volume of medical information may overload and confuse providers, particularly those that are not able to distinguish between reliable and poor-quality evidence. By engaging with CE that incorporates high-quality evidence, clinical practice guidelines, and systematic reviews, medical providers can update their care paradigms and improve patient outcomes [19]; indeed, in medical education, this has shown demonstrable improvements in both physician performance and patient health outcomes [20].

The above data suggest that allowing CE for activities that contribute to the research and academic pursuits of DCs will have a three-fold positive impact—it will contribute to the breadth of CE opportunities, facilitate evidence-informed practices in clinicians, all while simultaneously bolstering chiropractic academic and research development. As such, we propose that all states allow research and higher-education teaching activities for chiropractic CE credit to be consistent with that of ATs, DOs, and MDs CE requirements.

### Limitations

While we had at least two reviewers examine regulatory websites for each state it is possible that we misclassified states in terms of allowing CE credit for research or academic activities. None of the authors are legal experts, and legal jargon could have been misinterpreted. Many state board websites did not provide specific information on awarding CE for these activities. When a statute or regulation explicitly lists permitted items, legal canon indicates any unlisted items are typically understood to be excluded [21]. As such, any states that made no explicit statement regarding these activities were classified as not allowing CE for these activities. Additionally, there was an inconsistency where pertinent information was located on state board websites. Some states such as Ohio offer information regarding obtaining academic CE in a FAQ on their website. Other states provide this information within their regulatory language, while many states simply did not address research or academic CE.

## Conclusion

Chiropractors have significantly less opportunity to claim CE credit for research and higher-academic pursuits compared to ATs, PTs, MDs, and DOs. Precluding research and higher-education teaching opportunities for CE presents a barrier to the conduct and development of chiropractic research at present and in the future. State boards of chiropractic that do not currently allow for such CE opportunities are urged to promptly reconsider how both research and higher-education pursuits can magnify and advance the profession with vital knowledge development and adoption of evidence-based guidelines.

## Supplementary Information

Below is the link to the electronic supplementary material.

Supplementary Material 1

Supplementary Material 2

Supplementary Material 3

Supplementary Material 4

## Data Availability

No datasets were generated or analysed during the current study.
