# Correction: Continuing education for the chiropractic profession: a cross-sectional study analyzing potential barriers to future chiropractic academic and research development

**DOI:** 10.1186/s12998-025-00603-1

**Published:** 2025-09-16

**Authors:** Shannon Schueren, Dean L. Smith, Christopher A. Malaya, Jeffrey A. King, Nathan D. Schilaty

**Affiliations:** 1 Department of Kinesiology, University of Georgia, Athens, GA USA; 2 Department of Kinesiology, Nutrition and Health, Miami University, Oxford, OH USA; 3 Essence of Wellness Chiropractic Center, Eaton, OH USA; 4 Teaching, Learning, and Information Resources, Parker University, Dallas, TX USA; 5 Department of Neurosurgery, Medical College of Wisconsin, Milwaukee, WI USA; 6 Department of Neurosurgery, Brain, & Spine, University of South Florida, Tampa, FL USA; 7 Center for Neuromusculoskeletal Research, University of South Florida, Tampa, FL USA

**Correction to: Chiropractic & Manual Therapies (2025) 33:34** 10.1186/s12998-025-00596-x

The original publication of this article contained an incorrect Competing interests statement. The incorrect and correct information is shown in this correction article.

**Incorrect competing interests**

The authors declare no competing interests.

**Correct competing interests**

DLS: post-graduate online instructor for chirocredit.com, an online continuing education provider; JAK: occasional post-graduate instructor for CE programs.

